# Study on neuropathological mechanisms of primary monosymptomatic nocturnal enuresis in children using cerebral resting-state functional magnetic resonance imaging

**DOI:** 10.1038/s41598-019-55541-9

**Published:** 2019-12-16

**Authors:** Wen Zhu, Yingyu Che, Yan Wang, Zhiming Jia, Tingxiang Wan, Jianguo Wen, Jingliang Cheng, Chuanchuan Ren, Junwei Wu, Yunlong Li, Qingwei Wang

**Affiliations:** 1 Department of Urology, The First Affiliated Hospital of Zhengzhou University, Zhengzhou, Henan province 450052 China; 2 Department of Magnetic Resonance, The First Affiliated Hospital of Zhengzhou University, Zhengzhou, Henan province 450052 China; 3 Xinxiang Medical University, Xinxiang, Henan province 453003 China

**Keywords:** Urogenital diseases, Paediatrics, Urological manifestations

## Abstract

Primary monosymptomatic nocturnal enuresis (PMNE) is a heterogeneous disorder, which remains a difficult condition to manage due to lack of knowledge on the underlying pathophysiological mechanisms. Here we investigated the underlying neuropathological mechanisms of PMNE with functional MRI (fMRI), combining the amplitude of low frequency fluctuation (ALFF), regional homogeneity (ReHo), and seed-based functional connectivity (seed-based FC) analyses. Compared to the control group, PMNE group showed decreased ALFF value in the left medial orbital superior frontal gyrus (Frontal_Med_Orb_L), and increased ReHo value in the left superior occipital gyrus (Occipital_Sup_L). With left thalamus as the seed, PMNE group showed significantly decreased functional connectivity to the left medial superior frontal gyrus (Frontal_Sup_Medial_L). We conclude that these abnormal brain activities are probably important neuropathological mechanisms of PMNE in children. Furthermore, this study facilitated the understanding of underlying pathogenesis of PMNE and may provide an objective basis for the effective treatment.

## Introduction

Enuresis is a symptom of intermittent incontinence that occurs during sleep, requiring a minimum age of 5 years, a minimum of one episode per month, and a minimum duration of 3 months^1^. This disorder may continue into adolescence and could be a frustrating problem. Primary monosymptomatic nocturnal enuresis (PMNE) refers to the children who have never had a dry period of >6 months, without other lower urinary tract symptoms or bladder dysfunction. PMNE is a heterogeneous disorder, which remains a difficult condition to manage due to lack of knowledge on the underlying pathophysiological mechanisms. Genetics, nocturnal polyuria, nocturnal detrusor overactivity and disturbed central nervous system (CNS) mechanisms may be involved in the pathogenesis of PMNE^2–4^.

In recent years, functional MRI (fMRI) has provided an efficient, feasible, and non-radioactive method to investigate the brain activity. Using fMRI, blood oxygen level–dependent (BOLD) variation in the MRI signals associated with changes in neuronal activity of the brain is detected during rest or the performance of a task. BOLD-fMRI mainly includes resting-state fMRI (Rs-fMRI) and event-related fMRI. Rs-fMRI requires no explicit task performance during the examination, which is used to investigate changes in intrinsic brain activity^5^, while event-related fMRI needs to perform senior cognitive tasks^6^. Abnormality of intrinsic brain activity is thought to be correlated with developmental delay. Amplitude of low frequency fluctuation (ALFF) and regional homogeneity (ReHo) analyses are two common parameter analysis methods in Rs-fMRI studies^7^, while the former reflects the intrinsic brain activity in a single voxel level and the later reflects the synchronization activity between a given voxel and its neighborhood voxels. These two analysis methods have been widely applied in various diseases, such as Alzheimer’s disease, schizophrenia and depression^8–10^, to detect abnormal functional differences of brain regions or changes before and after treatment. To date, only one study applied ALFF and ReHo to evaluate enuresis related spontaneous brain activity changes^11^. However, in this study, the statistical analyses were uncorrected, which may result in false positive results. In addition, functional connectivity is also an efficient method to study brain global communication and function integration. Some previous studies showed that enuresis involved a consequence of abnormal functional connectivity rather than isolated brain regions^12,13^. In the lower urinary tract control system, the afferent impulses of bladder sensation and pontine micturition center (PMC) are transmitted through the thalamus to cerebral cortex^14^. Thus, thalamus is a highly integrated brain region associated with normal micturition. The features of functional connectivity of thalamus can be characterized using a seed-based correlation method with the voxels in the whole brain, which is called as seed-based functional connectivity (seed-based FC)^15,16^.

The objective of this study was to investigate the underlying neuropathological mechanisms of PMNE by combining ALFF, ReHo and thalamus seed-based FC, which may provide an objective basis for effective treatment.

## Subjects and Methods

### Ethical approval and informed consent

The study was approved by the ethics committee of the First Affiliated Hospital of Zhengzhou University (Zhengzhou, China, Ethic Number: 2017-KY-102). Methods were carried out in accordance with relevant guidelines and regulations. All subjects and their legal guardians were informed about the purpose of the study and provided written informed consent.

### Subjects

As for the exclusion and exclusion of the PMNE and control groups, a detailed case history, 3-day voiding diary, conventional urodynamics (CUD) and plain MRI scan were performed, respectively. A group of 37 right-handed children with PMNE (age: 11.3 ± 3.1 years, 20 males and 17 females) and a group of 17 right-handed control subjects (age: 11.1 ± 2.9 years, 9 males and 8 females) who needed surgery because of upper urinary tract diseases and with normal lower urinary tract function participated in this study. The intelligence quotient (IQ) of each participant was measured by trained professional staff using the Wechsler Intelligence Scale for Children Revised in China, and all participants possessed an IQ > 75. The presence of psychiatric diseases was excluded based on clinical examination according to the DSM-IV criteria and a structured history taking. In addition, the diagnosis of PMNE was strictly complied with the diagnostic criteria established by the International Children’s Continence Society (ICCS). Specifically, enuresis without other lower urinary tract symptoms (nocturia excluded), and without bladder dysfunction, is defined as monosymptomatic enuresis (MNE). PMNE is reserved for the children with MNE who have never had a previous dry period of >6 months. All of the children with PMNE in this study were treatment naive, and none of medical treatments including psychoactive drugs were administrated before fMRI study.

### Acquisition of Rs-fMRI images

All imageological examination was conducted at the Department of Magnetic Resonance (the First Affiliated Hospital of Zhengzhou University, Zhengzhou, China) on the Siemens 3.0 T Skyra MR system. All participants were asked to empty the bladder before the examination. Rs-fMRI requires no explicit task performance and all participants were asked to relax, keep eyes closed, and stay awake during the examination. Rs-fMRI images were acquired with a 12-channel phased array coil in the supine position. A total of 180 whole-brain volumes were scanned on 36 oblique slices (3 mm thick, interval = 1 mm) using a T2-weighted echo planar imaging sequence sensitive to BOLD contrast with the following parameters: repetition time (TR) = 2000 ms, echo time (TE) = 30 ms, flip angle = 90°, field of view (FOV) = 240 mm × 240 mm, matrix = 64 × 64.

### Data preprocessing

Data preprocessing was performed using DPABI software (http://rfmri.org/dpabi) based on Statistical Parametric Mapping 8 (SPM8, http://www.fil.ion.ucl.ac.uk/spm/) on the MATLAB platform (MathWorks, MA, USA). First, for each participant, the image quality was checked. Then, the first 10 time points were discarded to avoid the instability of the initial MRI signals and remaining Rs-fMRI images were converted to NIfTI files. Next, the fMRI data were corrected for the acquisition time delay and head motion. The head motion parameters of all participants were determined, and the inclusion criteria for head movement were <2.0 mm translation and <2° rotation during the fMRI scan. After these corrections, the images were directly normalized to the standard Montreal Neurological Institute (MNI) template at a 3 mm × 3 mm × 3 mm resolution and smoothed (except ReHo) with a Gaussian filter of FWHM = 6 mm × 6 mm × 6 mm. Finally, the resultant data were further filtered through a temporal band-pass (0.01–0.08 Hz) to avoid the interferences of low frequency drift and physiological noises.

### Calculation of ALFF, ReHo and seed-based FC

The ALFF was calculated using REST V1.8 (http://restfmri.net/forum/REST_V1.8) software. The time series were transformed to the frequency domain using fast Fourier transforms (FFTs) after preprocessing, and the power spectrum was obtained at each voxel. The square root of the power spectrum was calculated and then averaged across 0.01–0.08 Hz. The averaged square root was referred to as ALFF. Finally, each individual’s ALFF value was transformed to Z score to allow further comparison between groups.

Unsmoothed preprocessed data were applied to ReHo calculation using Kendall’s coefficient of concordance (KCC), which was applied to measure the synchronicity of the time series between a given voxel and its 26 nearest neighbors in a voxel-wise way. The generated ReHo data were subsequently smoothed with a Gaussian filter of FWHM = 6 mm × 6 mm × 6 mm.

The calculation of seed-based FC was also based on REST software. Thalamus is involved in mediation between PMC and cerebral cortex. Thus, left thalamus and right thalamus were chosen as the seeds to further explore the difference in functional connectivity between PMNE group and control group. Spherical seeds were defined with a radius of 6 mm, centered at the MNI coordinates of the left thalamus and right thalamus (left thalamus: −11, −18, 8; right thalamus: 13, −18, 8), respectively. The mean time series for each seed was computed separately for reference time course. Voxel-based general linear modeling (GLM) was applied to quantify the correlation between the seeds and other voxels in the whole-brain. The resulting r values were normalized to z values by Fisher’s z transform.

The resultant fMRI data were segmented into 116 brain regions using the automated anatomical labeling (AAL) atlas, which was widely applied in previous studies^15,17,18^. The AAL atlas incudes 90 regions in the cerebrum (45 in each hemisphere) and 26 regions in the cerebellum (9 in each hemisphere and 8 in the vermis).

### Statistical analysis

The data were expressed as the mean ± standard deviation (SD). Demographic data, including age, IQ, weight and bladder volume were compared between the PMNE and control groups using unpaired t-tests with SPSS 17.0 (IBM, USA) software. To compare the gender pro-frequency between the two groups, Chi-square test was used. Statistical tests were two-tailed, and *P* < 0.05 was considered significant for all the above mentioned statistical analyses. The data of ALFF, ReHo and seed-based FC were compared with unpaired t-tests using REST V1.8 software, with age and sex as covariates. The results were corrected for multiple comparisons to a significant level of *P* < 0.05 by AlphaSim correction combining individual voxel threshold *P* < 0.001 with a minimum cluster size >30 voxel. The results of ALFF and ReHo were visualized with REST Slice Viewer, while the results of seed-based FC were visualized with BrainNet Viewer. In addition, the data of ALFF and ReHo in significantly different brain regions between two groups were extracted and analyzed using SPSS 17.0 software, respectively. Unpaired t-tests were used and *P* < 0.001 was considered statistically significant.

## Results

### Demographic data

Participant demographics were summarized in Table 1. The two groups were matched for age and gender (PMNE group: age = 11.3 ± 3.1 years, 54% male participants; control group: age = 11.1 ± 2.9 years, 53% male participants). There was no significant difference in IQ between the PMNE and the control group (97.4 ± 7.2 vs. 96.8 ± 6.6, *P* > 0.05). In addition, the demographic data of weight and bladder volume also showed no significant differences between the two groups (*P* > 0.05). Detailed demographic data of the PMNE group and control group were listed (Supplementary Tables S1 and S2).

**Table 1:** Demographic data of the study participants.

	PMNE (n = 37)	Control (n = 17)	χ^2^ or t value	*P*
Age (years)	11.3 ± 3.1	11.1 ± 2.9	0.17	0.86
Male gender	20 (54%)	9 (53%)	0.01	0.94
IQ	97.4 ± 7.2	96.8 ± 6.6	0.30	0.77
Weight (Kg)	40.2 ± 7.8	43.8 ± 8.0	1.55	0.13
Bladder volume (ml)	362.5 ± 87.3	363.1 ± 80.7	0.02	0.98
Enuresis frequency (per week)	3.1 ± 1.5	NA	NA	NA

The data are presented as the mean ± SD. PMNE: primary monosymptomatic nocturnal enuresis; IQ: Intelligence quotient. *P < 0.05.

### ALFF and ReHo

**Figure 1:**
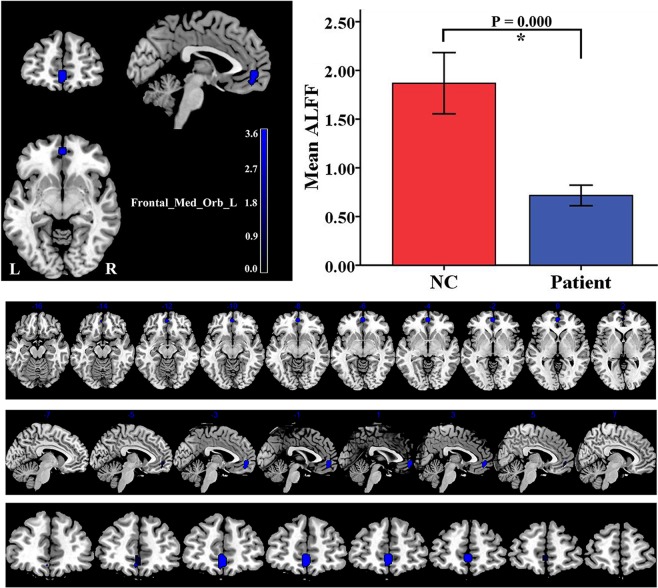
The PMNE group showed significantly decreased ALFF values in the Frontal_Med_Orb_L, compared to the control group. The data are expressed as the mean ± SD, and the error bars represent the SD. *P < 0.001. Frontal_Med_Orb_L: left medial orbital superior frontal gyrus; ALFF: amplitude of low frequency fluctuation; NC: normal control group; Patient: PMNE group.

**Figure 2:**
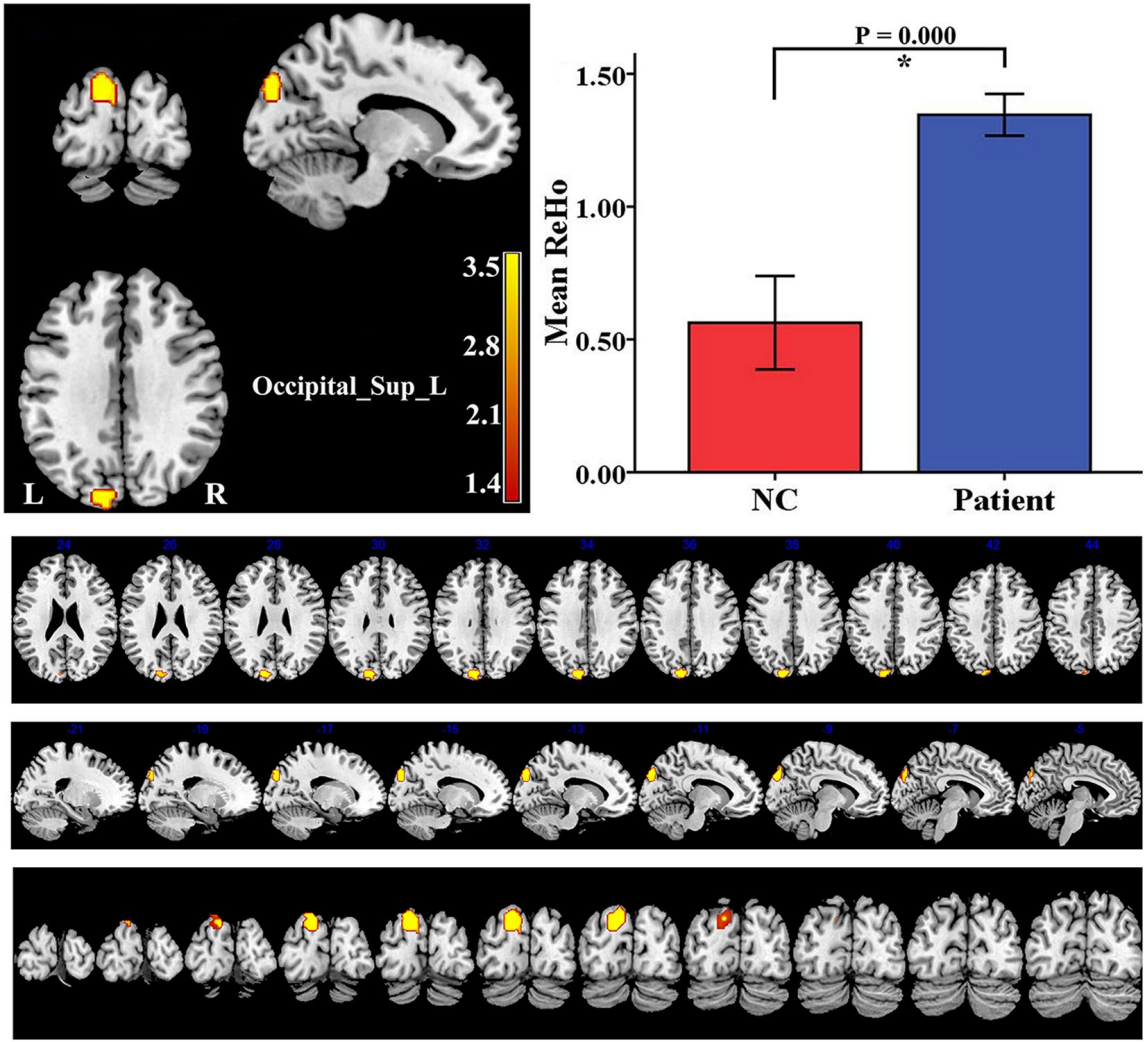
The PMNE group exhibited significantly increased ReHo values in the Occipital_Sup_L, compared to the control group. The data are expressed as the mean ± SD, and the error bars represent the SD. *P < 0.001. Occipital_Sup_L: left superior occipital gyrus; ReHo: regional homogeneity; NC: normal control group; Patient: PMNE group.

Compared to the control group, the PMNE group showed significantly decreased ALFF values in the left medial orbital superior frontal gyrus (Frontal_Med_Orb_L, AAL) (Fig. 1), and the details were shown in Table 2. In addition, the PMNE group exhibited significantly increased ReHo values in the left superior occipital gyrus (Occipital_Sup_L, AAL) (Fig. 2), and the details were shown in Table 2. The raw data of ALFF and ReHo extracted from the significantly different brain regions between the two groups were both shown as Supplementary Information Files (Supplementary Tables S3 and S4).

**Table 2:** BOLD signal changes in the PMNE group as compared to the control group.

	Increase/decrease	Brain region (AAL)	MNI coordinates			Peak t-value	Voxels
			X	Y	Z		
ALFF	Decrease	Frontal_Med_Orb_L	0	51	−3	−4.6	39
ReHo	Increase	Occipital_Sup_L	−15	−87	39	5.0	74

Results were corrected by AlphaSim. The clusters survived *P* < 0.05 by AlphaSim correction combining individual voxel threshold *P* < 0.001 with a minimum cluster size > 30 voxels for both ALFF and ReHo. The coordinates were showed in the Montreal Neurological Institute (MNI) standard space. BOLD: blood oxygenation level dependent; PMNE: primary monosymptomatic nocturnal enuresis; AAL: automated anatomical labeling; ALFF: amplitude of low frequency fluctuation; Frontal_Med_Orb_L: left medial orbital superior frontal gyrus; ReHo: regional homogeneity; Occipital_Sup_L: left superior occipital gyrus.

### Seed-based FC

**Figure 3:**
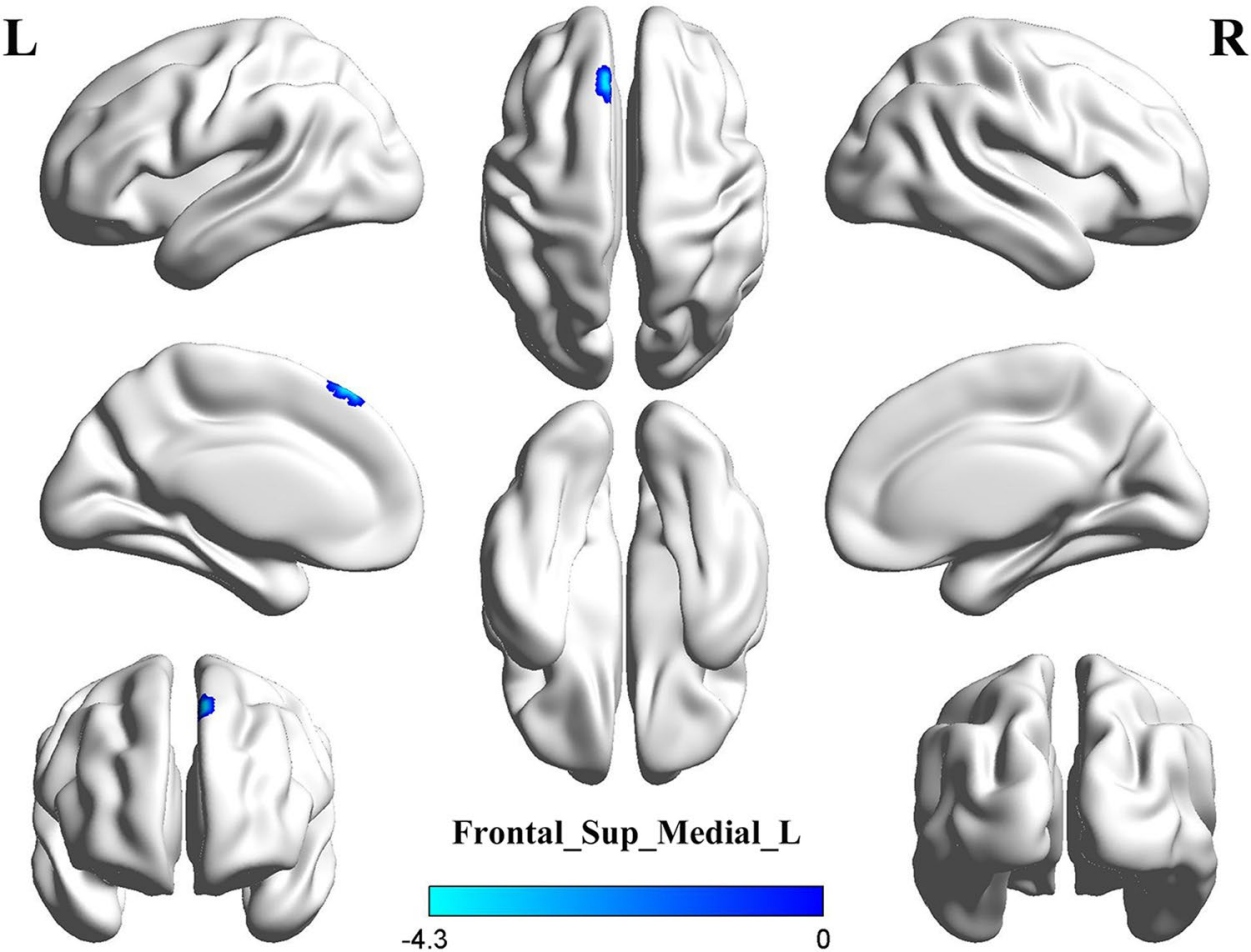
The PMNE group showed a significantly decreased functional connectivity between Frontal_Sup_Medial_L and the left thalamus (seed), compared to the control group. Frontal_Sup_Medial_L: left medial superior frontal gyrus.

The results of seed-based FC were shown in Fig. 3 and Table 3. With the seed located in the left thalamus, PMNE group showed significantly decreased functional connectivity to the left medial superior frontal gyrus (Frontal_Sup_Medial_L, AAL). While, with the seed located in the right thalamus, none of brain regions showed significantly different functional connectivity between the two groups.

**Table 3:** Altered functional connectivity in the PMNE group as compared to the control group.

Seed	Increase/decrease	Brain region (AAL)	MNI coordinates			Peak t-value	Voxels
			X	Y	Z		
Left thalamus	Decrease	Frontal_Sup_Medial_L	−6	36	60	−4.3	36

Results were corrected by AlphaSim. The clusters survived *P* < 0.05 by AlphaSim correction combining individual voxel threshold *P* < 0.001 with a minimum cluster size > 30 voxels for seed based functional connectivity. The coordinates were showed in the Montreal Neurological Institute (MNI) standard space. PMNE: primary monosymptomatic nocturnal enuresis; AAL: automated anatomical labeling; Frontal_Sup_Medial_L: left medial superior frontal gyrus.

## Discussion

The prevalence of PMNE has a range of 7.5–12.4% in children of 5–10 years and 1.6–5.3% in adolescents^19^. PMNE is often highly distressing for children and parents, and the quality of family life is affected. Moreover, loss of self-esteem, poor school performance, and domestic violence may occur. However, the etiology of PMNE is not completely clear, and the pathogenesis is mainly related to nocturnal polyuria, arousal disorder and developmental delay. Recently, the rapid development of fMRI paved a new way for the study on neuropathological mechanisms of PMNE.

Recently, the control center of urination has been roughly defined^14,20,21^. Bladder voiding is controlled not only by the midbrain and brainstem but also by superior cerebrum regions. The prominent cerebrum neural circuit involved in human voiding includes the thalamus, insula, lateral prefrontal cortex (LPFC), and medial prefrontal cortex (MPFC), as well as periaqueductal gray (PAG). Any dysfunction in these brain regions or abnormality of functional connectivity between two regions may result in deficient top-down control over the micturition reflex during sleep, which results in enuresis. Arousal disorder caused by the CNS also plays a vital role in the pathogenesis of PMNE^22^. The sleep-wake state of human is mainly regulated by the brainstem reticular formation, thalamus, posterior hypothalamus and basal forebrain^23^. If there is an abnormality in these brain areas, it may cause the child to have difficulty in awakening when the bladder reaches maximum capacity.

In this study, all fMRI examination was conducted with the Siemens 3.0 T Skyra MR system, and headphones or earplugs were given to the child and accompanying parent inside the imaging room to minimize the acoustic noise. As to the safety of fMRI for children, adverse cognitive or biological effects are not evident in the data from the longitudinal sample of children at the age range of 5 to 18 years during the course of 10 years of exposure to annual fMRI scan^24^. In addition, the U.S. Food and Drug Administration (FDA) considers a magnetic field safe up to 8 T for adults and children, and 4 T is considered safe for neonates^25^. In conclusion, 3.0 T fMRI can be considered safe for children. Although fMRI examination is a diagnostic tool, it should be used cautiously with the informed consent of patients and their legal guardians.

Rs-fMRI focuses on low-frequency oscillations in spontaneous brain activity, and ALFF and ReHo analyses are two common analysis methods used to investigate the characters of local intrinsic activity^7^. In this study, these two analysis methods were applied to study the neuropathological mechanisms of PMNE in children. The results indicated that the ALFF value of Frontal_Med_Orb_L decreased in the PMNE group, which was in accordance with the previous study^11^; ReHo value of Occipital_Sup_L increased, which was reported for the first time. Frontal_Med_Orb_L is located within MPFC, which is one of the most active brain regions in the frontal lobe. The MPFC communicates with the anterior cingulate gyrus, insula, thalamus and hypothalamus, which are all related to the control of voluntary micturition^14,26^. In addition, the prefrontal cortex (PFC) has been concerned with planning complex cognitive behaviors, moderating proper social behaviors and decision making. It was shown that a lesion of MPFC caused frequency and urgency of micturition when the patient is awake, incontinence when asleep. In this case, the sensation generating the desire to micturate is much diminished, and the sensation that micturition is imminent is also attenuated^27^. Another study revealed that children with PMNE showed abnormal gray matter volumes in MPFC and the supplementary motor area^28^. In conclusion, the Frontal_Med_Orb_L located in MPFC is most likely related to the pathogenetic mechanisms of PMNE; it can be suggested that the hypoactivity of this brain region may result in defective information gating and reduced inhibition of the micturition reflex during sleep, leading to enuresis.

The Occipital_Sup_L is a part of occipital lobe. Occipital lobe is the visual center of the brain, mainly responsible for the processing of visual information; it is also responsible for part of the language, action and abstract concepts and other functions. The occipital lobe injured or underdeveloped patients will not only show visual disorders, but may also appear to show memory loss or dyskinesia, and other symptoms. PMNE should be not regarded as a voiding disorder alone. One study showed that visuomotor integration ability was impaired to some extent in PNME children^29^, suggesting a possible correlation between the dysfunction of Occipital_Sup_L and PMNE. Focusing on the not-yet-understood relationship between the dysfunction of Occipital_Sup_L and PMNE could suggest a new perspective in the pathogenesis and management of pediatric PMNE.

The thalamus is a relay station which takes in the signals of sensation, perceptions and voluntary movements, and then passes these onto the cerebral cortex for further processing. Based on this, we set the left thalamus and right thalamus as seeds to detect the functional connectivity of CNS in children with PMNE. In the model of bladder control, it is currently accepted that the thalamus plays a vital role in relaying the signal from the bladder and PAG, and transmitting it to the insula, LPFC, MPFC in turn^14^. The activation of MPFC reduces the input to PAG, thus stabilizing the voiding reflex and maintaining continence. Frontal_Sup_Medial_L which is adjacent to Frontal_Med_Orb_L is also located in the MPFC. The significantly decreased functional connectivity between the thalamus and Frontal_Sup_Medial_L may affect the thalamic neuronal signal transmission to MPFC. If this occurs, we suppose that the reduction of the input to PAG is not enough to restrain the voiding reflex during sleep, leading to bedwetting. This point is in accordance with the observation that the MPFC is deactivated in response to bladder filling in patients with urgency incontinence^30^. The thalamus also plays a vital role in regulating the state of sleep or arousal^31,32^. As children with PMNE urinate voluntarily during the daytime, the pathogenesis of PMNE is not just an abnormality of micturition reflex but a disorder of sleep. Previous research suggested that thalamocortical oscillations contributed to switching between the aroused state and the sleep state^33^. Therefore, the decreased functional connectivity between the thalamus and MPFC may also affect the ability of children with PMNE to wake up because of the sensation of a full bladder.

In this study, all dysfunctional brain regions were detected in the left hemisphere, which seemed to be in contradiction with the previous view that brain regions which control micturition are predominantly in the right hemisphere^34^. The left hemisphere may act as an assistant role in the control of voiding. We assumed that the left hemisphere of children with PMNE possibly cannot control voiding properly in nature. However, this disorder could be compensated by the normal function of right hemisphere in the daytime when they are awake but could not during sleep, leading to bedwetting.

### Limitations

Although this study is conducive to reveal the neuropathological mechanisms of PMNE, some limitations still exist. Rs-fMRI cannot be completed under the status of sleep at night because of the noise during the examination. Therefore, it is less physical to reflect the true situation of the children wetting at night. In addition, although the results showed the ReHo value in the Occipital_Sup_L is abnormal in the PMNE group, the in-depth relationship of the Occipital_Sup_L and micturition control is unclear, which needs studies with better methods of brain function research to clarify. Finally, the sample size of this study is relatively small, and we did not go further into the comparison of different age groups of children with PMNE. We will continue to collect more clinical cases for further comparative studies.

## Conclusion

The dysfunction of the Frontal_Med_Orb_L and Occipital_Sup_L, decreased functional connectivity between Frontal_Sup_Medial_L and the left thalamus are possibly main neuropathological mechanisms of PMNE in children. This study defined the dysfunctional brain regions which involve in the onset of PMNE and may provide an objective basis for the effective treatment.

## Supplementary information

Supplementary information

## Data Availability

The datasets generated during and/or analyzed during the current study are available in the figshare repository, https://figshare.com/s/c0ad2d5dc195cbe6470d; 10.6084/m9.figshare.10334927.
