# Supplementary material for: Study on neuropathological mechanisms of primary monosymptomatic nocturnal enuresis in children using cerebral resting-state functional magnetic resonance imaging
Source: Sci Rep. 2019 Dec 16;9:19141. doi: 10.1038/s41598-019-55541-9 (PMC6915704; doi:10.1038/s41598-019-55541-9)
Supplement: Supplementary file 1 — Supplementary information [file 41598_2019_55541_MOESM1_ESM.pdf]

Study on neuropathological mechanisms of primary monosymptomatic nocturnal enuresis in children using cerebral resting-state functional magnetic resonance imaging

Wen Zhu[1,#], Yingyu Che[2,#], Yan Wang[1,#], Zhiming Jia[1], Tingxiang Wan[1], Jianguo Wen[1,3], Jingliang Cheng[2], Chuanchuan Ren[1], Junwei Wu[1], Yunlong Li[1] & Qingwei Wang[1,*]

[1]Department of Urology, The First Affiliated Hospital of Zhengzhou University, Zhengzhou, Henan province, 450052, China

[2]Department of Magnetic Resonance, The First Affiliated Hospital of Zhengzhou University, Zhengzhou, Henan province, 450052, China

[3]Xinxiang Medical University, Xinxiang, Henan province, 453003, China

[#]These authors contributed equally to this work.

[*]Corresponding author. Correspondence and requests for materials should be addressed to Q.W. (email: qwwang@zzu.edu.cn)

Supplementary Table S1

No.	Age (years)	Gender	Intelligence quotient (IQ)	Weight (Kg)	Enuresis frequency (per week)	Bladder volume (ml)	Frequency of waking up for voluntary voiding
1	14	Male	98	45	2-3	460	Sometimes
2	13	Female	97	40	3	405	Sometimes
3	12	Female	105	42	2	410	Often
4	8	Female	84	37	4	263	Often
5	11	Male	92	39	2-4	350	Often
6	8	Male	96	33	5	285	Never
7	9	Female	89	40	3	305	Never
8	12	Male	98	37	2	387	Often
9	15	Male	106	39	1-2	470	Often
10	15	Male	94	50	3	485	Sometimes
11	13	Female	102	48	7	409	Never
12	12	Female	100	35	3-4	395	Often
13	14	Male	97	39	3	460	Often
14	8	Male	91	34	2	258	Never
15	14	Male	104	45	2-3	435	Sometimes
16	13	Female	93	41	3	392	Often
17	7	Female	87	26	4	235	Often
18	13	Female	109	39	4-5	405	Sometimes
19	14	Male	103	46	7	412	Sometimes
20	12	Male	107	40	2	371	Sometimes

21	7	Male	92	26	1	225	Never
22	8	Female	95	40	2	263	Never
23	9	Male	101	31	2-3	305	Sometimes
24	7	Female	90	29	3	250	Often
25	8	Male	85	42	5	275	Sometimes
26	15	Male	99	50	2-3	486	Often
27	14	Female	106	42	3	431	Often
28	16	Female	93	51	1	452	Often
29	7	Male	91	29	2	263	Sometimes
30	9	Female	99	34	5	280	Often
31	8	Male	103	39	3-4	292	Sometimes
32	15	Male	110	53	2	471	Never
33	8	Female	86	40	2-3	255	Never
34	9	Male	94	30	3	286	Sometimes
35	16	Male	108	54	1	504	Sometimes
36	15	Female	107	58	7	466	Often
37	9	Female	92	46	3	317	Sometimes

Supplementary Table S1: Demographic data of the PMNE group. All patients wet bed only at night.

Supplementary Table S2

No.	Age (years)	Gender	Intelligence quotient (IQ)	Weight (Kg)	Bladder volume (ml)
1	7	Male	93	30	245
2	12	Male	95	43	402
3	9	Female	106	37	315
4	8	Female	101	35	268
5	14	Male	110	48	440
6	13	Male	93	50	417
7	9	Female	87	39	305
8	15	Male	99	54	464
9	13	Female	103	46	410
10	12	Male	100	50	387
11	16	Male	98	56	479
12	13	Female	94	53	430
13	10	Male	102	38	318
14	7	Female	90	34	245
15	8	Male	85	40	276
16	14	Female	97	53	455
17	9	Female	92	39	316

Supplementary Table S2: Demographic data of the control group.

Supplementary Table S3

PMNE group (No.)	Mean ALFF	Control group (No.)	Mean ALFF
1	0.74	1	2.12
2	0.50	2	1.61
3	0.72	3	1.87
4	0.71	4	1.65
5	0.93	5	2.35
6	0.80	6	1.84
7	0.82	7	2.15
8	0.79	8	1.57
9	0.64	9	1.90
10	0.76	10	1.58
11	0.80	11	1.50
12	0.66	12	1.71
13	0.62	13	2.42
14	0.79	14	2.12
15	0.51	15	1.64
16	0.76	16	2.29
17	0.45	17	1.43
18	0.70		
19	0.84		
20	0.90		
21	0.81		
22	0.85		
23	0.57		
24	0.55		
25	0.82		

26	0.72
27	0.64
28	0.60
29	0.73
30	0.74
31	0.82
32	0.87
33	0.72
34	0.79
35	0.66
36	0.70
37	0.61

Supplementary Table S3: The data of mean ALFF extracted from the significantly different brain region between the two groups.

Supplementary Table S4

PMNE group (No.)	Mean ReHo	Control group (No.)	Mean ReHo
1	1.39	1	0.41
2	1.24	2	0.49
3	1.35	3	0.58
4	1.32	4	0.63
5	1.37	5	0.58
6	1.36	6	0.82
7	1.42	7	0.39
8	1.25	8	0.50
9	1.31	9	0.59
10	1.32	10	0.19
11	1.33	11	0.65
12	1.47	12	0.80
13	1.52	13	0.33
14	1.13	14	0.72
15	1.53	15	0.79
16	1.41	16	0.73
17	1.34	17	0.55
18	1.40		
19	1.42		
20	1.32		
21	1.40		
22	1.37		
23	1.40		
24	1.41		
25	1.50		

26	1.21
27	1.29
28	1.41
29	1.36
30	1.37
31	1.36
32	1.46
33	1.34
34	1.15
35	1.40
36	1.35
37	1.31

Supplementary Table S4: The data of mean ReHo extracted from the significantly different brain region between the two groups.
